# Individually-tailored multifactorial intervention to reduce falls in the Malaysian Falls Assessment and Intervention Trial (MyFAIT): A randomized controlled trial

**DOI:** 10.1371/journal.pone.0199219

**Published:** 2018-08-03

**Authors:** Pey June Tan, Ee Ming Khoo, Karuthan Chinna, Nor I’zzati Saedon, Mohd Idzwan Zakaria, Ahmad Zulkarnain Ahmad Zahedi, Norlina Ramli, Nurliza Khalidin, Mazlina Mazlan, Kok Han Chee, Imran Zainal Abidin, Nemala Nalathamby, Sumaiyah Mat, Mohamad Hasif Jaafar, Hui Min Khor, Norfazilah Mohamad Khannas, Lokman Abdul Majid, Kit Mun Tan, Ai-Vyrn Chin, Shahrul Bahyah Kamaruzzaman, Philip Poi, Karen Morgan, Keith D. Hill, Lynette MacKenzie, Maw Pin Tan

**Affiliations:** 1 Ageing and Age-Associated Disorders Research Group, University of Malaya, Kuala Lumpur, Malaysia; 2 Geriatric Education and Research Institute, Singapore; 3 Department of Primary Care Medicine, Faculty of Medicine, University of Malaya, Kuala Lumpur, Malaysia; 4 Department of Social and Preventive Medicine, Faculty of Medicine, University of Malaya, Kuala Lumpur, Malaysia; 5 Division of Geriatric Medicine, Department of Medicine, Faculty of Medicine, University of Malaya, Kuala Lumpur, Malaysia; 6 Department of Trauma and Emergency Medicine, Faculty of Medicine, University of Malaya, Kuala Lumpur, Malaysia; 7 Department of Ophthalmology, Faculty of Medicine, University of Malaya, Kuala Lumpur, Malaysia; 8 Department of Rehabilitation Medicine, Faculty of Medicine, University of Malaya, Kuala Limpur, Malaysia; 9 Division of Cardiology, Department of Medicine, Faculty of Medicine, University of Malaya, Kuala Lumpur, Malaysia; 10 Department of Rehabilitation Medicine, Kuala Lumpur, University of Malaya Medical Centre, Kuala Limpur, Malaysia; 11 Department Psychology and Behavioural Science, Perdana University-RCSI School of Medicine, Serdang, Selangor, Malaysia; 12 School of Physiotherapy and Exercise Science, Faculty of Health Sciences, Curtin University, Perth, Australia; 13 Department of Occupational Therapy, Faculty of Health Sciences, University of Sydney, Sydney, Australia; University of Glasgow, UNITED KINGDOM

## Abstract

**Objective:**

To determine the effectiveness of an individually-tailored multifactorial intervention in reducing falls among at risk older adult fallers in a multi-ethnic, middle-income nation in South-East Asia.

**Design:**

Pragmatic, randomized-controlled trial.

**Setting:**

Emergency room, medical outpatient and primary care clinic in a teaching hospital in Kuala Lumpur, Malaysia.

**Participants:**

Individuals aged 65 years and above with two or more falls or one injurious fall in the past 12 months.

**Intervention:**

Individually-tailored interventions, included a modified Otago exercise programme, HOMEFAST home hazards modification, visual intervention, cardiovascular intervention, medication review and falls education, was compared against a control group involving conventional treatment.

**Primary and secondary outcome measures:**

The primary outcome was any fall recurrence at 12-month follow-up. Secondary outcomes were rate of fall and time to first fall.

**Results:**

Two hundred and sixty-eight participants (mean age 75.3 ±7.2 SD years, 67% women) were randomized to multifactorial intervention (n = 134) or convention treatment (n = 134). All participants in the intervention group received medication review and falls education, 92 (68%) were prescribed Otago exercises, 86 (64%) visual intervention, 64 (47%) home hazards modification and 51 (38%) cardiovascular intervention. Fall recurrence did not differ between intervention and control groups at 12-months [Risk Ratio, RR = 1.037 (95% CI 0.613–1.753)]. Rate of fall [RR = 1.155 (95% CI 0.846–1.576], time to first fall [Hazard Ratio, HR = 0.948 (95% CI 0.782–1.522)] and mortality rate [RR = 0.896 (95% CI 0.335–2.400)] did not differ between groups.

**Conclusion:**

Individually-tailored multifactorial intervention was ineffective as a strategy to reduce falls. Future research efforts are now required to develop culturally-appropriate and affordable methods of addressing this increasingly prominent public health issue in middle-income nations.

**Trial registration:**

ISRCTN Registry no. ISRCTN11674947

## Introduction

Asian nations and other developing countries worldwide currently experiencing population ageing at a faster rate than that experienced by North America and Western Europe in the previous century [1]. Falls in older persons will, therefore, inevitably pose a large and growing burden to healthcare services in these countries. Falls are common among older adults and are associated with adverse physical consequences including hip fractures, other major fractures and intracranial bleeding [2, 3]. Functional dependency and psychological consequences associated with falls such as depression and pathological fear-of-falling have also been observed after an index fall [4]. In addition, one in four older people who present to emergency services following a fall were found to be no longer alive after one year [5].

Numerous intervention studies have been conducted for primary and secondary prevention of falls in older adults to address this serious public health issue [6]. While multi-factorial interventions have been shown to be beneficial as a secondary prevention strategy to reduce frequency of falls, available evidence have been drawn from predominantly Caucasian populations, with few falls studies conducted in Asia to date [7]. A recent scoping review found no published randomized controlled studies using multifactorial interventions for secondary falls prevention in South-East Asia, though a handful of studies have now been registered [8]. Transferability of fall prevention approaches to local contexts is challenging, with limited existing infrastructure for geriatric care, which is largely unstructured, with an estimated doctor-to-patient ratio of one geriatrician per 100,000 older persons in Malaysia [9]. Malaysia’s multi-ethnic community also contributes to unique cultural differences which may influence fall risk factors [10].

The effect of multicomponent interventions is also considered variable, with many negative studies in existence despite pooled results suggesting overall benefit [11–14]. Therefore, available evidence on the efficacy of multifactorial interventions remains conflicting, and may be dependent upon the combination of the component interventions as well as local variations in care infrastructure [15]. We aimed to determine the effectiveness of an individually-tailored multifactorial intervention in secondary falls prevention among at risk older adult fallers in a multi-ethnic, middle-income nation in South-East Asia, to address this gap in the literature.

## Materials and methods

This was a pragmatic, parallel randomized-controlled trial using a simple randomization with a 1:1 allocation ratio. The study protocol has been registered as a clinical trial (clinical trial registration number: ISRCTN11674947) S1 Text and the full protocol is published elsewhere [16]. A brief description of the study protocol is provided here. The recruitment period was from 2012 to 2014, with the last participant’s follow-up visit occurring in February 2016. Recruitment was stopped once an adequate sample sizes was obtained. The study was approved by the local institutional review board, University of Malaya Medical Ethics Committee (ethics number 925.4), and written informed consent was obtained from all participants.

Community-dwelling individuals aged 65 years and older with a history of two or more falls or one injurious fall over the past 12 months were recruited from the emergency department, medical outpatients and primary care clinic at a teaching hospital in Kuala Lumpur, Malaysia. The exclusion criteria were: clinically-diagnosed dementia, major psychiatric illnesses and inability to stand. Eligible, consenting participants were randomized to individually-tailored multifactorial intervention or conventional treatment, with general health advice given to all participants. Randomization was performed using a computer generated random number sequence, and treatment allocation was concealed using sealed-opaque envelopes and stored in a secure location. Enrolment and assessments were conducted by trained research assistants. Fall recurrence were monitored through monthly fall diaries. Participants were re-assessed after 12 months at a hospital visit.

### Baseline assessment

All information was collected using a computerised data collection instrument. Baseline information on age, sex, ethnicity, social circumstances, medical history and medications were obtained at enrolment. A detailed falls history for the preceding 12 months including mechanism of falls, number of falls, associated injury, medical attention received and presence of any associated symptoms were also obtained. Following that, all participants were assessed using standardized assessment tools to identify potential risk factors for falls: gait and balance, visual impairment, falls risk medications, cardiovascular risk, fear-of-falling and depression. Our assessors consisted of geriatricians, psychiatrists, an ophthalmologist, a physiotherapist, a rheumatologist and research assistants who have received training in the administration of all assessment items.

#### Physical performance assessment

Gait and balance were assessed with the Timed-Up and Go test (TUG) and functional reach (FR) [17, 18]. The TUG test score was considered the time taken by participants to rise from a chair with armrests, walk forward three metres at their usual pace, turn around and return to the chair. Functional reach was measured by asking the participants to stand with their feet together, their left shoulder next to a metre ruler fixed on a wall with their left arm outstretched, and to lean forwards as far as possible without losing their balance. The maximal difference between the initial ruler measurement at the fifth metacarpal head with the participant upright and leaning forward was recorded. Hand-grip strength was measured using a Jamar dynamometer with the participant seated upright and elbow at 90 degrees of flexion.

#### Visual assessment

Visual acuity was assessed using the Snellen chart. Visual acuity was then converted to LogMAR values for subsequent analysis. Contrast sensitivity was assessed bilaterally using the Pelli-Robson chart at one-metre distance and stereopsis assessed with the Frisby Near Stereoacuity test. The tumbling E chart was used for those who were unable to recognize alphabets [19].

#### Cardiovascular assessment

All participants were assessed with a 12-lead electrocardiogram (ECG), and lying and standing oscillometric blood pressure, which was measured first in the supine position, and at 1, 2 and 3 minutes of standing after 10-minutes’ rest in the supine position. Continuous beat-to-beat blood pressure was also used to record blood pressure changes from the supine to erect posture throughout (Task Force® Monitor, CNSystems, Austria). A systolic and/or diastolic blood pressure drop of more than 20 and/or 10 mmHg respectively was considered significant for the diagnosis of orthostatic hypotension [20].

#### Psychological assessments

Fear-of-falling was determined using the short 7-item Falls Efficacy Scale-International (short FES-I) [21]. This instrument was translated into the Malay language (the national language of Malaysia) and validated, and previously validated versions in Chinese and English were also validated prior to being administered to our participants. A short FES-I score of 11 or more out of a maximum score of 28 was considered fear-of-falling [22]. Depression, anxiety and stress were determined using the 21-item Depression, Anxiety and Stress Scale (DASS-21) [23]. This tool had previously been validated in all three languages–English, Malay and Mandarin. The pre-determined cut-offs for the presence of depression, anxiety and stress were 7, 6 and 10 respectively [24]. Individuals with severe and extremely severe anxiety and depression were referred for psychological follow-up for ethical reasons, and not included as part of this study.

### Individually-tailored multifactorial interventions

Individuals randomized to the intervention arm were prescribed up to six components of the multifactorial intervention. A modified Otago exercise programme, visual intervention, home environmental modification, medication review and cardiovascular intervention were provided according to the outcome of their baseline assessments based on predefined criteria. All participants received falls education and medication review. The appropriate interventions were assigned following the assessment by a geriatrician.

#### Modified Otago exercise programme

Individuals with either a TUG score of 13.5 seconds or greater, FR of less than 18 centimetres, clinical evidence of gait and balance disorders or fear-of-falling were referred for physical intervention using a modified Otago programme [17, 18, 25]. The original Otago programme was modified by removing the walking component and replacing home visits with hospital-based assessments. The programme was administered by a trained physiotherapist. Participants were asked to attend the hospital geriatric rehabilitation gym on four occasions: at baseline and monthly for three months. Each participant was given a training pack which included pictorial instructions for their exercises, a training diary which they were required to complete and a pair of 500g ankle weights. Participants were first assessed for their ability by the physiotherapist at baseline and prescribed exercises with at appropriate the intensity. They were then instructed to perform these exercises daily for at least five times per week, which consisted of an individually-tailored combination of five to eight strength and balance exercises. The intensity of exercises was adjusted accordingly at subsequent follow-up visits, and at the final visit, they were encouraged to continue with their exercises as much as possible and to participate in Tai Chi and other group-based exercises in their communities. Results from the modified Otago Exercise Programme has been published elsewhere [26, 27].

#### Visual interventions

Individuals with a Snellen visual acuity of 6/12 or poorer in one or both eyes were referred to an ophthalmologist for further assessment. Detailed ophthalmic examinations were carried out and interventions were prescribed accordingly. These included spectacle prescriptions for significant refractive errors, lubricants for symptomatic dry eyes, initiation of glaucoma medications and early cataract surgery [28].

#### Home environment modification

Individuals with at least one indoor fall which was non-syncopal in nature and who agreed to be visited by an occupational therapist were referred for home hazards assessment. This was performed by a trained occupational therapist using the Home Falls and Accidents Screening Tool (HOME FAST) [29]. The HOMEFAST is a 25-item checklist used to systematically report potential hazards in and around the home. Recommendations were made by the occupational therapist based on the hazards identified. To facilitate immediate implementation, aids and equipment were provided and installed upon completion of the environmental assessment where feasible. The modifications provided focused on improving movement and night time visibility such as adhesive glow tapes at corners, light switches and steps of staircases.

#### Medication review

All participants were asked to bring both their medication prescription list and all their medications to the initial appointment. The information was corroborated with hospital electronic prescribing records and additional confirmation was obtained through follow-up telephone calls to participants if necessary. This medication list was reconciled by a trained medical practitioner and reviewed by a trained geriatrician. Falls risk increasing drugs were identified and discontinued whenever possible [30]. If the participant’s medical condition did not allow for drug discontinuation, dose-reduction or replacement with newer more selective drugs were organized (e.g. a selective alpha-1-adrenoreceptor agonist for a non-selective alpha-adrenoreceptor agonist for benign prostatic hypertrophy). Decisions for medication changes were made by the attending geriatrician based on clinical judgement using the above strategy.

#### Cardiovascular interventions

The initial cardiovascular assessments were reviewed alongside the falls history by the geriatrician. Individuals with an abnormal 12-lead ECG were further investigated with echocardiography and ambulatory ECG monitoring. Individuals with a history of unexplained falls with no evidence of cardiac syncope or clinically significant orthostatic hypotension were then assessed with carotid sinus massage and tilt-table testing using previously published protocols [31, 32]. Permanent cardiac pacemakers were implanted for those with evidence of bradyarrhythmia on ambulatory monitoring or 12-lead ECG. Those with evidence of ischaemia were referred to a cardiologist for further assessment. Individuals with symptomatic or large postural blood pressure changes were given conservative advice and had potential culprit medications discontinued as described in the section above.

#### Falls education

Falls education was provided using printed material explaining common risk factors and how to avoid future falls, alongside verbal advice as per individual established risks. Attending family members were also counselled with regards to falls risks and prevention where possible. Otherwise the participants were encouraged to read through the printed materials with their spouses and adult children. The printed information were sent out by post to participants with their second set of falls diaries.

### Outcome measures

The primary outcome was the difference in proportion of participants experiencing a fall during the 12-month follow-up period between the intervention and control groups; while the secondary outcome was the difference in rate of falls between the two groups. The first fall event occurring during the treatment period was considered the primary outcome. ‘Rate of fall’ was defined as the number of fall events occurring over the 12-month treatment period. Time to first fall and mortality was also compared between groups as secondary outcomes. Falls were recorded prospectively using monthly fall diaries with daily entries. These diaries were available in three languages (English, Malay and Mandarin) and used pictorial representations. Participants and attending family caregivers were instructed on how to complete these diaries at the end of their baseline assessment visit and providing with their first month’s falls diary. Monthly diaries were sent out by post with self-addressed stamped envelopes over the subsequent 11 months. Diary returns were encouraged with reminder telephone calls to participants who did not return their diaries three months in a row. Participants who received diaries but did not return them by post as they had no further falls, were recorded as ‘not returned as no falls’ after this was confirmed through a telephone call.

### Data analysis

The sample size estimated has been published previously [16]. In brief, assuming 50% of fallers experience fall recurrence without intervention, 93 participants in each group will provide 80% power to detect a reduction in fall occurrence in the intervention group to 30% using the Chi-squared test to the significance level of 0.05. The sample size was extended to 133 participants per group to allow for a liberal dropout rate which is expected in studies involving frail older individuals. Analyses of falls outcomes were conducted on an intention-to-treat basis on all randomised individuals including those who had died, withdrew consent or were lost to follow up. Baseline comparisons were presented as mean with standard deviations and frequencies with percentages for continuous and categorical variables respectively. Outcomes were compared with binary logistic regression and negative binomial regression, presented as odds ratios (OR) and rate ratios (RR) with 95% confidence intervals (CI). Time to first fall was compared using Cox-proportional hazards analysis which allowed for different lengths of follow-up, hazards ratios (HR) with 95% CI are presented for this. All significant differences are taken at p≤0.05 level. Sensitivity testing was performed to determine the best method of for imputation of missing data. Missing diaried falls data were replaced using a combination of multiple imputations (five times) and linear interpolation sorted according to age to maintain the power level and control for Monte Carlo errors [33]. Imputed diaried falls was triangulated with self-reported falls at follow-up to obtain total number of falls. Participants who were completely lost-to-follow up (no diary and self-reported falls data) and died before completion of 12-month follow-up were assumed to have no falls. Total number of falls was treated with the same method of imputation to obtain final total number of falls. No between-group hypothesis testing was conducted for baseline characteristics and no adjustments were made for baseline differences as the sample was randomized and hence any difference was assumed to have occurred by chance [34]. Analysis was conducted using the Statistical Package for Social Sciences (SPSS) version 20.

## Results

**Fig 1:**
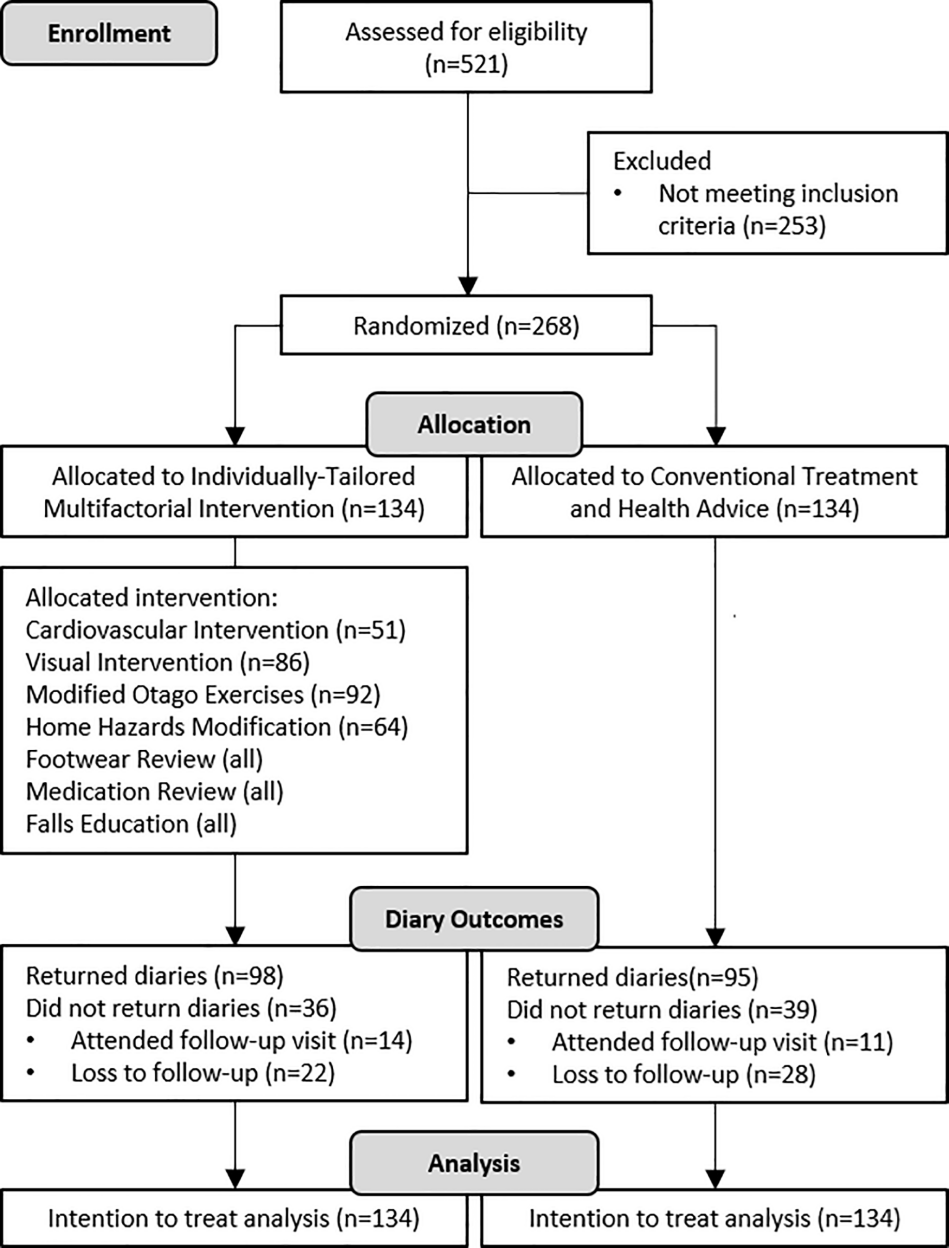
CONSORT flow diagram of recruitment, treatment allocation and follow-up.

Two hundred and sixty-eight participants were recruited. Fig 1 presents the CONSORT flow diagram S2 Text, which describes the recruitment process, treatment allocation and attrition. The participants’ mean age was 75.3 (±7.2 SD) years and 67% (n = 176) were female. The ethnic distribution was 61% (n = 166) Chinese, 17% (n = 45) Malay, 19% (n = 51) Indian and 2% (n = 6) others. Table 1 summarizes the baseline characteristics of participants in both intervention and control groups. Among participants who were randomized to the intervention group, 92 (68%) were prescribed modified Otago exercises, 86 (64%) visual intervention, 64 (47%) home hazard modification and 51 (38%) cardiovascular interventions. Table 2 summarizes the list of probable risk factors identified following the initial assessment, prior to further home hazards evaluation using the HOMEFAST and confirmatory cardiovascular assessments.

**Table 1:** Baseline characteristics of participants.

Characteristics	Intervention (n = 134)	Control (n = 134)
Age, mean (SD)	74.5 (6.8)	76.1 (7.5)
Female, n (%)	93 (69.4)	88 (65.7)
Ethnicity, n (%)		
*Malay*	24 (17.9)	21 (15.5)
*Chinese*	76 (56.7)	90 (67.2)
*Indian*	30 (22.4)	21 (15.7)
*Others*	4 (3.0)	2 (1.5)
Waist hip ratio, mean (SD)	0.88 (0.08)	0.89 (0.07)
Total comorbidities, median (range)	2 (0–8)	3 (0–10)
BMI, mean (SD)	24.5 (4.2)	23.9 (4.1)
Number of medications, median (range)	4 (0–13)	4 (0–21)
TUG (second), mean (SD)	17.7 (12.7)	17.7 (11.02)
FR (centimeter), mean (SD)	23.5 (8.04)	23.2 (8.5)
Short FES-I, mean (SD)	14.3 (6.1)	13.4 (5.6)
Depression, mean (SD)	7.3 (8.7)	7.1 (8.7)
Stress, mean (SD)	8.1 (8.1)	7.3 (7.4)
Anxiety, mean (SD)	4.0 (4.5)	4.0 (5.3)
Unexplained falls, n (%)	49 (36.6)	52 (38.8)
Injury from falls, n (%)	100 (74.6)	95 (70.9)

*SD* Standard deviation, *BMI* Body mass index, *TUG* Timed-up and Go, *FR* Functional reach, *FES-I* Falls efficacy scale international.

**Table 2:** Risk factors identified in all participants at baseline.

Risk Factor	No. Participants (n = 268)	%
≥1 Fall risk increasing drug	204	76
Fear of falling (Short FES-I>11)	177	66
Gait and balance abnormalities	158	60
Orthostatic hypotension	103	59
Visual disorder	134	50
Depression (DASS-21 Depression>7)	102	38
Suspected home hazards*	82	31
Anxiety (DASS-21 Anxiety>6)	75	28
Arthritis	69	26
Visual impairment	65	25
Vasovagal syncope*	61	23
Poor footwear	52	17
Osteoarthritis	48	18
Stress (DASS-21 Stress>10)	27	10
Foot problems	27	10
Hearing impairment	30	11
Situational syncope	22	8
Peripheral neuropathy	18	7
Incontinence	10	4
Spinal problems	10	4
Stroke disease	8	3
Parkinson’s disease	6	2

* Clinically diagnosed ± Tilt-table test. FES-I = Falls Efficacy Scale-International, DASS-21 = 21-item Depression, Anxiety and Stress Scale.

Note: Based on clinical assessment and participant self-report at baseline assessment.

### Falls outcomes

**Fig 2:**
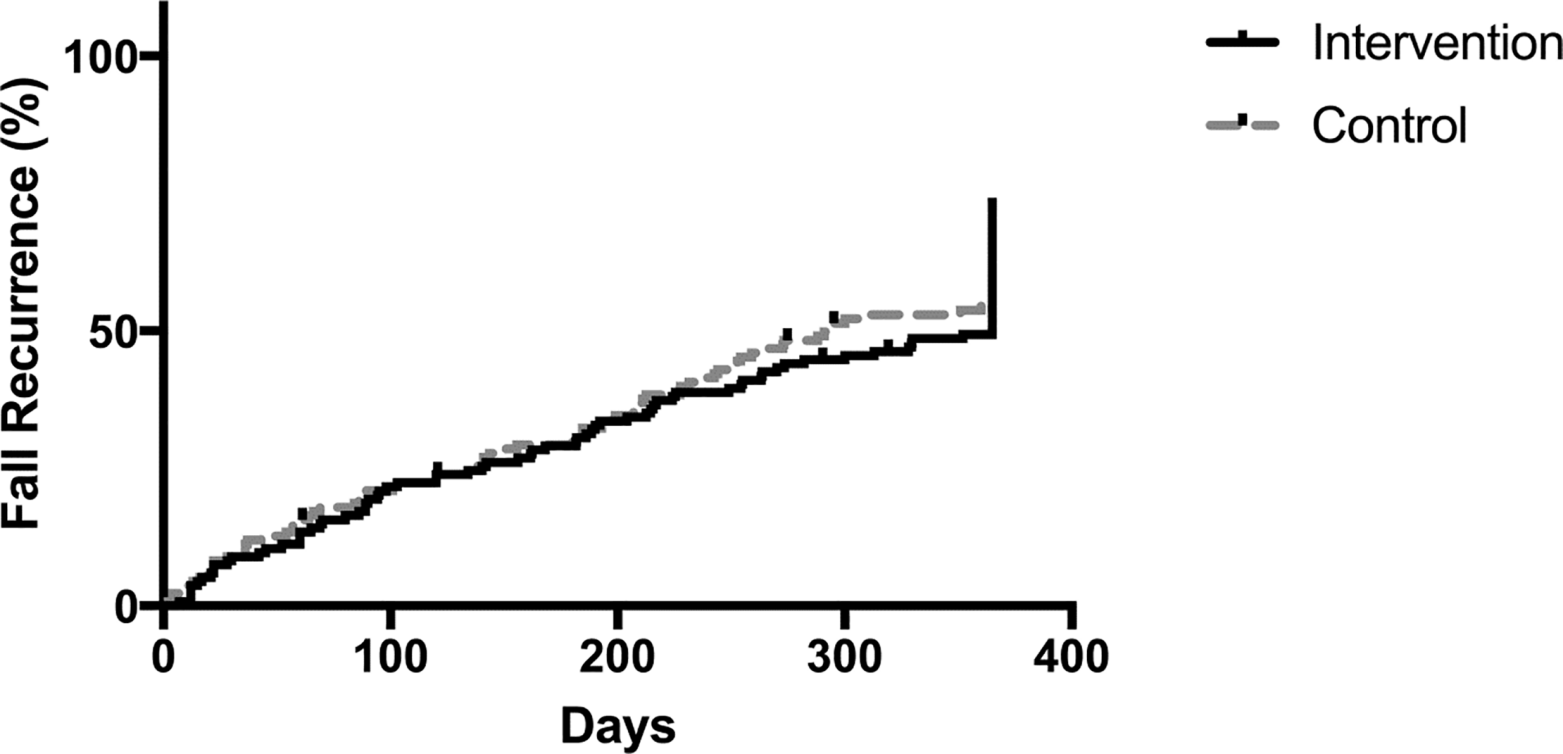
Cumulative hazard plot for time to first fall among participants in the intervention and control groups.

A total of 506 fall events were documented. Fig 2 illustrates the cumulative falls recurrence over time for both the intervention and the control groups. Ninety-five (70.5%) participants in the intervention group and 94 (70.1%) participants in the control group reported at least one fall in the 12-month follow-up period. No significant differences were found in fall recurrence and mortality between groups (Table 3).

**Table 3:** Results at 12-months follow-up.

Characteristics	n	Intervention	n	Control	Ratio (95% CI)	p-value
Number of falls, n	134	274	134	232		
Rate of fall, mean (SD) *	134	2.0 (2.3)	134	1.8 (1.7)	RR 1.16 (0.85–1.58)	0.38
Fall recurrence, n (%) ‡	134	95 (70.5)	134	94 (70.1)	OR 1.04 (0.61–1.75)	0.89
Mortality, n (%) ‡	129	8 (6.2)	131	9 (6.9)	OR 0.90 (0.34–2.40)	0.83
Time to first fall (days), mean (SD) §	134	255.3 (129.9)	134	243.1 (131.5)	HR 0.95 (0.71–1.26)	0.71

* Negative binomial regression, RR Rate Ratio

‡ Binary logistic regression, OR Odds Ratio

§ Cox regression, HR Hazard Risk Ratio

Note: Missing data in mortality as old identification number was not found in national database.

The estimated mean number of falls per person was 1.9 (±2.1 SD). The intervention group had a higher rate of falls (274 falls, 2.0 falls per person, ±2.3 SD) compared to the control group (232 falls, 1.8 falls per person, ±1.7 SD). However, there was no significant difference in the rate of falls between intervention and control groups (RR = 1.16; 95% CI 0.85 to 1.58).

The estimated mean time to first fall was 249.2 (±130.6 SD) days. The intervention group had a lower cumulative hazard risk (255.3 days ±129.9 SD) compared to the control group (243.1 days ±131.5 SD). However, there was no significant difference in time to first fall (days) between intervention and control groups (HR = 0.95, 95% CI 0.71 to 1.26).

## Discussion

This randomized-controlled study which compared individually-tailored multifactorial interventions to conventional treatment among older adults with recurrent or injurious falls in a middle-income, South-East Asian nation had found no difference in the fall recurrence, rate of fall and time to first fall between intervention and control groups at 12-month follow-up. This was a pragmatic study which uniquely evaluated a complex intervention in a developing country setting where the healthcare system remains hospital centric with minimal primary healthcare and community health support [35].

Malaysia is considered an upper middle-income developing nation by the United Nations situated at the equator. Malaysia’s multi-ethnic community comprises of majority Malays followed by Chinese and Indians, with individual groups preserving their ancestral cultures and lifestyles [36]. A study involving complex interventions for older adults in this setting is therefore likely to expose important issues surrounding the Asian culture and health beliefs that may influence the delivery of such interventions, which would be relevant to the highly populous continent of Asia that consists of 62% of the world’s 7 billion people [37].

The mean age of participants in our study was comparable to previous randomized controlled studies evaluating multifactorial interventions. However, mortality rate of our sample population was slightly higher than that reported in previous studies, with a 6.3% attrition due to all-cause mortality compared to 2 to 5% in previous community-based samples [38]. This suggests that the included sample was potentially more frail with similar characteristics to those who we are likely to treat in our clinics [39]. This suggests that we had been inclusive in our criteria and fall definition [40]. A pragmatic design was adopted to avoid the pitfalls of previous clinical trials involving treatments for age-associated conditions which had evaluated interventions among populations which are not reflective of real-world conditions [41]. Hence, pragmatic designs and minimal exclusions, such as that reported in the present study, should be encouraged in future studies to ensure the results of clinical trials are applicable in the real-world.

Our fall recurrence rate of 70% does reflect that reported by other studies, which reported a similar recurrence rate among at-risk individuals with at least one fall over the previous 12-months [11, 38, 42], confirming the validity of our diary exercise. Pooled analysis of previous multifactorial interventions had demonstrated benefits in number of falls rather than fall recurrence [43]. However, only a handful of multifactorial intervention studies were performed on at-risk community-dwelling individuals as indicated by our inclusion criteria. The lack of standardization in the use of fall terminology and operationalization, choice of target population and reporting techniques have led to challenges in comparison of outcomes [40, 44]. In addition, few multifactorial falls intervention studies have reported time to first fall as an outcome. This additional approach was selected to allow for the variable nature of fall events and different lengths of follow-up due to the expected high attrition rate. However, as the delivery of multiple interventions are likely to require time to administer and for treatment benefits to take effect, time to first fall measured over the first 12-months after recruitment, may not yield positive results. Extending the period of follow-up may not necessarily be beneficial due to our high fall prevalence in both arms [40].

The total number and type of interventions in a multifactorial program is likely to contribute to the net effect on falls reduction in our study. Campbell and Robertson have previously suggested that compounding interventions could lead to a decrease in measured (or unmeasured) advantages of a multifactorial intervention program due to intervention-to-intervention interaction [15]. A number of single interventions were found to be effective in falls reduction [45]. However, this positive effect may be neutralized when applied in tandem with another intervention. An Australian study with a 3-intervention model (exercise program, home modification and visual intervention) found an added reduction in the rate of falls for every additional intervention [46]. Comparatively, a Taiwanese 5-intervention model found no significant effect on falls incidence [47]. The MyFAIT study used a 6-intervention in its approach [48] which may potentially lead to more complex intervention-to-intervention interaction. It could be possible that two or more interventions may lead to more change than the older person is comfortable with accepting [15].

Adherence issues were observed in many multifactorial intervention studies including the MyFAIT study [15, 46, 47]. The delivery of multifactorial interventions involves numerous challenges that may be primarily attributed to both participant and staff adherence. Older individuals in Malaysia are often dependent on their adult children and are unable to attend hospital appointments unless driven by their adult children to hospital, due to the poor public transport system and poor disabled access for all public areas including hospitals [49]. Due to limitations in elderly-friendly infrastructure and hospital services, attending a hospital appointment often requires the older adult to be accompanied by at least two other family members. While all initial assessments including medical review were conducted in one visit, further cardiovascular investigations, ophthalmology assessments and physiotherapy sessions all required additional hospital visits. Previous studies have reported challenges in the translation of multifactorial interventions to clinical settings [50]. This could be largely due to older adults’ attitudes towards fall risks in terms of not seeing falls as a problem. Especially in the uptake of home modifications, one study identified an unanticipated lack of interest in modifying their residences due to a home-self relationship [51]. This was followed by limited freedom to carry out home adaptations as many of our older adults lived in homes owned by their adult children [52]. However, our findings should be interpreted with caution as it is only applicable to urban, community-dwelling older adults aged 65 years and above residing in Kuala Lumpur and is therefore non-generalizable. Further exploration of adherence issues relating to the mix of interventions trialled in the MyFAIT study, in a developing Asian country context are warranted.

Falls recurrence may not necessarily be the most useful outcome to measure in all fall intervention studies. An incident fall may be the indication of the older adult developing physical frailty or a marker of cognitive decline. In closely knit societies with a strong emphasis on family values such as in Asian societies, older adults may either restrict their own activities or have activity restriction enforced by their families after a fall event, with any loss of function that may ensue being seamlessly absorbed by family caregivers [53]. Therefore, the measurement of fall outcomes alone may not necessarily reveal any significant difference perhaps due to the differences in fall avoidance strategies and potentially higher levels of supervision afforded through extended family households and larger family sizes. Future analyses should consider evaluating the relative contribution of each component intervention as well as explore more appropriate outcome measures for falls interventions among older fallers in our setting.

## Conclusion

No reduction in fall recurrence, rate of fall or time to first fall were observed over a 12-month follow-up period in a randomized-controlled study comparing individually-tailored multifactorial interventions to conventional treatment as secondary falls prevention. Future studies should consider evaluating in greater detail, cultural differences in behaviour and outcomes following fall events, as well as identify culturally appropriate and affordable solutions for the management of high-risk fallers in lower and middle-income countries.

## Supporting information

S1 Text Study protocol. Registered protocol of the MyFAIT study.
(PDF)

S2 Text CONSORT checklist. Completed CONSORT 2010 checklist of information to include when reporting a randomised trial for the MyFAIT study.
(PDF)
