# Supplementary material for: Individually-tailored multifactorial intervention to reduce falls in the Malaysian Falls Assessment and Intervention Trial (MyFAIT): A randomized controlled trial
Source: PLoS One. 2018 Aug 3;13(8):e0199219. doi: 10.1371/journal.pone.0199219 (PMC6075745; doi:10.1371/journal.pone.0199219)
Supplement: S1 Text — Registered protocol of the MyFAIT study. (PDF) [file pone.0199219.s001.pdf]

Website: http://www.isrctn.com/ISRCTN11674947

ISRCTN11674947 DOI 10.1186/ISRCTN11674947

THE MALAYSIAN FALLS ASSESSMENT AND INTERVENTION TRIAL

Condition category

Injury, Occupational Diseases, Poisoning

Date applied

27/10/2012

Date assigned

27/11/2012

Last edited

26/05/2017

Prospective/Retrospective

Retrospectively registered

Overall trial status

Completed

Recruitment status

No longer recruiting

Plain English Summary

Background and study aims
About one in three older people over the age of 65 years fall at least once a year. Falling over often leads to loss of confidence and avoidance of activity. Some older people are admitted to nursing homes because of repeated falls. Falls can also lead to severe health problems which include fractures of the hip bone and bleeding in the brain. The older person often becomes very dependent suffering the above consequences. Falls in the older person tend to be due to a few problems rather than just one problem on its own. These problems include heart problems, low blood pressure, side-effects of medications, poor balance, muscle weakness, previous strokes, eyesight and the home environment. To treat falls in the older person, we need to look into the whole person and deal with all the potentially treatable causes. The purpose of this study is to find out whether a treatment package which looks into treating multiple possible conditions that increase the risk of falls is an effective method of treating falls in the older population in Malaysia.

Who can participate?
Patients with one fall resulting in physical injuries or two or more falls over the past 12 months from the primary care clinic, geriatric clinic and Accident and Emergency.

What does the study involve?
The patients will be approached by a researcher to find out if they are interested in the study. Those who agree to take part will then be given an appointment to attend a falls clinic. At the falls clinic, patients will be given a medical check-up to find out what may be causing them to fall. This will involve taking a history, a physical examination, an ECG heart trace, blood pressure and eye tests The doctor may also order some blood tests, X-rays and some other tests if required. We will also be asking questions about the psychological effects of falls. After the medical check-up, patients will be assigned to two groups: the regular treatment or the active intervention group. The regular treatment group will continue to receive the treatment planned by the doctors they first saw at the clinic or A&E. They will asked to fill in a falls diary and the researcher will contact them regularly over the next 12 months to find out how they are, and they will be seen again in 12 months for a check-up. If the patient is assigned to the active intervention group, they will be given individually-tailored treatment, which will be prescribed by the doctor. This can include changes to their medication, heart tests, physiotherapy, and a home visit. The doctor may also refer the patient to the heart specialist, eye specialist or ENT specialist. If the doctor finds that the patient has a balance problem, he/she will be referred for physiotherapy, which will involve exercises to strengthen his/her muscles. If the patient had a fall in their own home, we will ask an occupational therapist to visit them at home to see if we can help make their home safer.

What are the possible benefits and risks of participating?
Participants will receive a health check at no charge, and any medical problems identified during health screening will be addressed as appropriate. There are no known risks associated with participating in this trial.

Where is the study run from?
University of Malaya Medical Centre (Malaysia)

When is the study starting and how long is it expected to run for?
July 2012 to February 2016

Who is funding the study?
University of Malaya (Malaysia)

Who is the main contact?
Dr Maw Pin Tan
mptan@ummc.edu.my

Trial website

Contact information

Type

Scientific

Primary contact

Dr Maw Pin Tan

ORCID ID

Contact details

Department of Medicine

Faculty of Medicine

University of Malaya

Kuala Lumpur

50603

Malaysia

Additional identifiers

EudraCT number

ClinicalTrials.gov number

Protocol/serial number

N/A

Study information

Scientific title

The Malaysian Falls Assessment and Intervention Trial: a randomized controlled trial

Acronym

MyFAIT

Study hypothesis

Multifaceted interventions is effective in reducing falls in older Malaysians with a history of previous falls.

Ethics approval

Medical Ethics Committee, University of Malaya Medical Centre, 20/06/2012, ref: 925.4

Study design

Single-centre randomized controlled trial

Primary study design

Interventional

Secondary study design

Randomised controlled trial

Trial setting

GP practices

Trial type

Quality of life

Patient information sheet

Not available in web format, please contact Dr M P Tan, mptan@ummc.edu.my to request a patient information sheet

Condition

Falls in older people

Intervention

Individually-tailored, multifaceted interventions involving modifiable risk factors of falls:
1. Cardiovascular Assessment and Intervention
2. Medication Review
3. Physiotherapy Prescribed Strength and Balance Exercise Programme
4. Home-hazards Intervention
5. Visual assessment and intervention
6. Others- as required

Intervention type

Other

Phase

Not Applicable

Drug names

Primary outcome measures

Fall recurrence over 12 months follow-up measured with a falls diary. Participants will also receive telephone interviews every 2 months to corroborate the accuracy of the diaries and to improve compliance.

Secondary outcome measures

Autonomic measures, gait and balance measures and psychological measures conducted on initial assessment. Follow up visits to be hospital will be conducted at 12 months.

Overall trial start date

01/07/2012

Overall trial end date

02/02/2016

Reason abandoned

Eligibility

Participant inclusion criteria

1. Individuals aged >65 years
2. Two or more falls or one injurious fall over the past 12 months

Participant type

Patient

Age group

Senior

Gender

Both

Target number of participants

300

Participant exclusion criteria

1. Clinical diagnosis of dementia
2. Inability to stand

Recruitment start date

10/07/2012

Recruitment end date

23/12/2014

Locations

Countries of recruitment

Malaysia

Trial participating centre

University of Malaya
Kuala Lumpur
50603
Malaysia

Sponsor information

Organisation

University of Malaya Medical Centre (Malaysia)

Sponsor details

c/o Prof Dato Ikram Shah bin Ismail
Lembah Pantai
Kuala Lumpur
59100
Malaysia

Sponsor type

University/education

Website

http://www.ummc.edu.my

Funders

Funder type

University/education

Funder name

University of Malaya (Malaysia) ref: RP-010-2012

Alternative name(s)

University of Malaya, UM

Funding Body Type

government organisation

Funding Body Subtype

government non-federal

Location

Malaysia

Results and Publications

Publication and dissemination plan

Not provided at time of registration

Intention to publish date

Participant level data

Not provided at time of registration

Results - basic reporting

Publication summary

2014 protocol in: http://www.ncbi.nlm.nih.gov/pubmed/24951180

Publication citations

1. Protocol
   Tan PJ, Khoo EM, Chinna K, Hill KD, Poi PJ, Tan MP, An individually-tailored multifactorial intervention program for older fallers in a middle-income developing country: Malaysian Falls Assessment and Intervention Trial (MyFAIT)., BMC Geriatr, 2014, 14, 78, doi: 10.1186/1471-2318-14-78.

- PubMed Abstract
  - Publisher Full Text

Additional files

Editorial Notes

26/05/2017: The following changes were made to the trial record: 1. The overall trial start date was changed from 01/11/2012 to 01/07/2012. 2. The overall trial end date was changed fr
